# Design, synthesis, and enzyme inhibition evaluation of some novel Mono- and Di-*O*-β-D-Glycopyranosyl Chalcone analogues with molecular docking studies

**DOI:** 10.55730/1300-0527.3527

**Published:** 2022-11-23

**Authors:** Gonca ÇELİK, Gizem TATAR YILMAZ, Hüseyin SAHİN, Burak BARUT, Nurettin YAYLI

**Affiliations:** 1 Department of Chemistry, Faculty of Science, Karadeniz Technical University, Trabzon, Turkey; 2 Department of Biostatistics and Medical Informatics, Faculty of Medicine, Karadeniz Technical University, Trabzon, Turkey; 3 Espiye Vocational School, Giresun University, Giresun, Turkey; 4 Department of Biochemistry, Faculty of Pharmacy, Karadeniz Technical University, Trabzon, Turkey; 5 Department of Pharmacognosy, Faculty of Pharmacy, Karadeniz Technical University, Trabzon, Turkey

**Keywords:** Chalcone, enzyme inhibition, glycoside, glycosylation, molecular docking

## Abstract

In this study, some novel mono- and di-*O*-β-D-glycopyranosyl chalcone analogs were designed, synthesized, and characterized. The chalcone derivatives were synthesized with good yields by base-catalyzed Claisen-Schmidt condensation in EtOH solution. Then these chalcones were reacted with TAGBr (2,3,4,6-tetra-O-acetyl-α-D-glucopyranosylbromide) in dry acetone under the anhydrous condition at 0–5 °C. Deacylated was carried out by the Zemplen’s method with NaOCH_3_ in dry methanol results in substituted chalcone-*O*-glycosides (mono- and di-*O*-β-D-glycopyranosyl chalcone analogs). The chemical structures of all synthesized compounds were elucidated based on IR, NMR spectral data, and mass spectrometry. Further, the compounds (**7a–c, 8a–c, 12a–c, 16a–c,** and **17a–c**) were tested for their enzyme inhibition activity against α-glycosidase, tyrosinase, and AChE with *in vitro* and *in silico* analysis. Amongst them, compounds **12a–c, 16a–c,** and **17a–c** displayed moderate or less enzyme inhibition activity against α-glycosidase while other compounds **7a–c** and **8a–c**) were not active. Remarkably interesting enzyme inhibition effects, with IC_50_ values below 30.59 ± 0.30 μM were recorded with **7c** (IC_50_=11.07 ± 0.55 μM) against tyrosinase.

## 1. Introduction

Chalcones (1,3-diaryl-2-propen-1-one) consisting of two aromatic rings are joined by three carbons and one carbonyl in which is α, β-unsaturated carbonyl system [1]. Chalcones and their derivatives represent an essential group of heterocyclic compounds due to their excellent skeletons [2]. Synthetic or naturally occurring chalcone derivatives describe as target molecules for the drug design and development because of their wide range of pharmacological properties, including antiinflammatory, antibacterial, anti-Alzheimer, and anticancer [3].

**Scheme 1:**
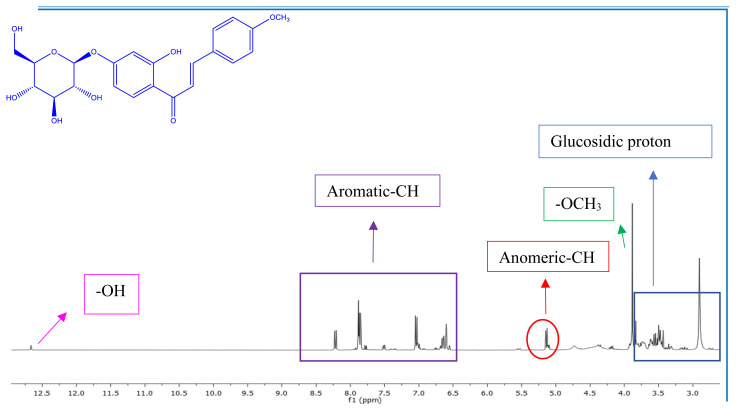
Structure of isolated chalcone glycosides (**1–3**) from *B. rapa* L. ‘hidabeni’.

Glycosylation (mono, di, and tri) modification could greatly amend the water solubility, instability, and biological properties [4,5]. When chalcone derivatives flanked by bulky sugar groups, chalcone glycosylated derivatives formed at the positions *O-*Aryl or *C-*Aryl [6,7]. In nature, particularly glycoside moiety like chalcone glycoside forms are widely exist as β-configuration. In addition, previous studies revealed that chalcones are generally found as chalcone-*O*-glycosides that were extensively explored among their derivatives in natural products [8]. For instance, Ninomiya and co-workers reported that three new chalcone glycosides such as 4′-*O*-β-D-glucopyranosyl-4-hydroxy-3′-methoxychalcone (**1**), 4′-*O*-β-D-glucopyranosyl-3′,4-dimethoxychalcone (**2**), and 4,4′-di-*O*-β-D-glucopyranosyl-3′-methoxychalcone (**3**) were isolated from *Brassica rapa* L. ‘hidabeni’ and their chemical structure was identified using spectroscopic methods (Scheme 1) [9]. In addition, a literature survey reported that dihydrochalcones (DHCs) glycosides had shown significant antioxidant capacities [10]. Moreover, it was reported that chalcone glycosides affected NO production in microglia HAPI cells [11]. However, in contrast to an extensive isolation study on chalcone-*O*-glycosides from plants, few studies have been demonstrated the synthesis of their derivatives containing an *O*-glycosides structure [12].

One of the common endocrine diseases in the world is diabetes mellitus (DM). And it is thought that its prevalence will reach nearly 366 million by 2030 [13,14]. The imbalance of blood sugar absorption, insulin secretion, and insulin action can be seen as the common results of this endocrine disease [15]. According to the general knowledge, two types of diabetes were defined because of having different pathogenesis. Except for type 1, the treatment of type 2 is focused on therapeutic applications, especially one of them is based on the α-glycosidase inhibition summarized as the blocking of degradation of oligo- and disaccharides [13]. Extensively known α-glycosidase inhibitors (AGIs) such as Acarbose, Voglibose, and Miglitol [16] are proven their efficiency in the inhibition capacity of α-glycosidase. As in all pharmacological studies, scientific studies also related to the inhibition of α-glycosidase are still going on to find the best potential drug by having the least side effect.

Tyrosinase (EC 1.14.18.1) is known as a multifunctional copper-containing oxidase enzyme, which has a significant function in melanin biosynthesis and hydroxylation of monophenols molecules in animals, plants, fungi, and bacteria [17,18]. The melanin is liable for the color of human skin, animal fur, and plant browning which guards the skin against injury caused by ultraviolet (UV) light in sunlight [19]. It has been reported that the excessive reproduction of melanin results in some pigmentation diseases, such as freckles, acne, melasma, pregnancy spots in females, malignant melanoma, senile lentigines, solar lentigo, and parkinson [20–22]. Therefore, tyrosinase inhibitors (hydroquinone, arbutine, kojic acid, corticosteroids) have happened a significant target for researches in as well as cosmetics and pharmaceutical industries [23,24]. Akhtar et al. [25] reported that some chalcone derivatives could be possible tyrosinase inhibitors. Additionally, 1-(2-cyclohexylmethoxy-6-hydroxy-phenyl)-3-(4-hydroxymethyl-phenyl)-propenone exhibited a strong tyrosinase activity and inhibition of melanin production [26]. Although most literature was performed on the chalcone and their derivatives tyrosinase inhibition activity, there is a very limited study about chalcone-*O*-glycosides tyrosinase inhibitory activities.

Acetylcholinesterase (AChE, EC 3.1.1.7) hydrolyzes the neurotransmitter acetylcholine to finish nerve impulses these enzymes affect synaptic junctions [27,28]. Some recent studies show that the lack of AChE begins with the loss of memory and learning impairment of Alzheimer’s disease (AD) cases [29]. Moreover, the rate of AD in the aged population which is risen quickly and is currently a worldwide health problem. In addition to this, AChE inhibitors treated to mild to moderate AD such as physostigmine, rivastigmine, tacrine, donepezil, and galantine [30]. Kızıltas et al. [31] revealed that galantamine is currently an important potential inhibition compound from crude extracts for the treatment of AD. Liu et al. [32] revealed that the chalcone derivatives, which contained an especially dimethylamino group exhibited significantly AChE inhibitory effect. According to Zhang et al. [33] chalcones along with their derivatives play beneficial roles to treat deadly AD. However, there is no literature report that the chalcone-*O*-glycosides acetylcholinesterase inhibitory activities that could be potential drugs for treatment AD had been studied yet.

The main aims of this study are to synthesize and characterize mono- and di-glycoside conjugates of some novel chalcone-*O*-glycosides as well as to evaluate their enzyme (α-glycosidase, tyrosinase, and AChE) inhibition activity by experimental and binding mechanism by molecular docking studies. We hope this work could pave the route for the synthesis of new chalcone-*O*-glycosides, searching for new agents for the pharmaceutical industry.

## 2. Experimental

### 2.1. Materials and methods

^1^H NMR and ^13^C NMR spectra were recorded with a Bruker Avance instrument at 400 and 100 MHz at room temperature. Here, the acetone-*d*_6_, methanol-*d*_4_, and dimethyl sulfoxide-*d*_6_ were selected as the NMR solvent and δ ppm against Me_4_Si (TMS) as internal standards, respectively. High-resolution mass spectrometry was measured using Micromass Quattro LC-MS/MS and Agilent 1260 Infinity Series LC/Q-TOF. The IR spectra were measured with a Perkin Elmer 1600 Fourier transform infrared (FTIR-ATR) spectrophotometer in the range of 4000–400 (cm^−1^) and expressed in wavenumber (cm^−1^). Melting points of all compounds were determined using a Thermo-var apparatus fitted with a microscope in degrees (°C) and are uncorrected. Reaction progress was checked by TLC (thin layer chromatography, Merck) using aluminum silica gel plates 60 F_254_. Camag UV-Vis spectrophotometer was recorded the UV spectra at 366–254 nm and spray with 10% aqueous H_2_SO_4_ solution and heating. All reagents and solvents were directly purchased from the Merck and Sigma-Aldrich chemical industries.

#### 2.1.1. General procedure for the synthesis of compounds 6a**–**c, 11a**–**c, 15c

**Scheme 2:**
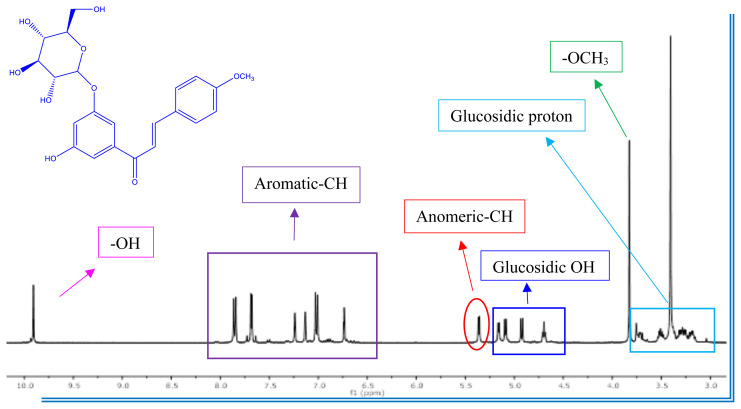
The chemical structure of target molecule and synthetic route of **4–17 (a-c).**

The studied compounds are illustrated in Scheme 2. Compounds **6a–c, 11a–c,** and **15c** were prepared according to the previously described procedures [12,34].

#### 2.1.2. The general method for the synthesis of 15a and 15b

A mixture of 3,5-dihydroxy acetophenone (**13**, 0.01 mol), substituted benzaldehyde (**14a** and **14b**, 0.01 mol) in aqueous potassium hydroxide (KOH) (20 mL, 40%), and ethanol (40 mL) were stirred at room temperature. After stirring for approximately 20 h, the reaction mixture was checked by thin-layer chromatography (TLC) with silica gel (60 F_254_) plates. The reaction mixture was poured into 200 mL ice-cold water to obtain a solid product. The resulting solid was filtered off, washed with water to remove potassium hydroxide, and dried. It was recrystallized from ethanol to give compounds **15a** and **15b** in good yields.

##### 2.1.2.1. (2*E*)-1-(3,5-dihydroxyphenyl)-3-(2-methoxyphenyl)prop-2-en-1-one (15a)

Yield: 55%, m.p.: 72–74 °C. IR *v_maks_*(cm^−1^): 3261 (-OH), 3078 (aromatic C-H), 2944 (aliphatic C-H), 1644 (C=O), 1599, 1563, 1448 (C=C), 1246, 1018 (C-O), 746.^1^H NMR (400 MHz, Acetone-*d*_6_, ppm) δ 7.45 (d, *J* = 15.7 Hz, 1H, H-2, A part of AB system), 8.10 (d, *J* = 15.7 Hz, 1H, H-3, B part of AB system), 6.93 (d, *J* = 2.8 Hz, 1H, H-2′), 6.42 (dd, *J* = 7.0/2.8 Hz, 1H, H-4′), 6.92 (d, *J* = 2.8 Hz, 1H, H-6′), 7.18 (d, *J* = 7.6 Hz, 1H, H-3″), 7.02 (t, *J* = 7.6 Hz, 1H, H-4″), 7.24 (t, *J* = 7.8 Hz, 1H, H-5″), 7.6 (d, *J* = 7.8 Hz, 1H, H-6″), 3.86 (s, 3H, 2″-OCH_3_).^13^C NMR (100 MHz, Acetone-*d*_6_, ppm) δ 189.7 (C-1), 122.7 (C-2), 140.2 (C-3), 132.6 (C-1′), 106.5 (C-2′), 159.0 (C-3′), 106.8 (C-4′), 159.0 (C-5′), 106.5 (C-6′), 129.2 (C-1″), 158.7 (C-2″), 112.2 (C-3″), 132.6 (C-4″), 121.2 (C-5″), 123.4 (C-6″), 56.1 (2″-OCH_3_). HRMS (ESI, *m/z*) for C_16_H_14_O_4_ [M+H]^+^: calcd 270.2799, found 271.0942 (95).

##### 2.1.2.2. (2*E*)-1-(3,5-dihydroxyphenyl)-3-(3-methoxyphenyl)prop-2-en-1-one (15b)

Yield: 50%, m.p.: 66–68 °C. IR *v_maks_*(cm^−1^): 3387 (-OH), 3011 (aromatic C-H), 2949 (aliphatic C-H), 1630 (C=O), 1594, 1451 (C=C), 1234 (C-O), 972, 857, 760. ^1^H NMR (400 MHz, Acetone-*d*_6_, ppm) δ 7.30 (d, *J* = 15.7 Hz, 1H, H-2, A part of AB system), 7.62 (d, *J* = 15.7 Hz, 1H, H-3, B part of AB system), 6.96 (s, 1H, H-2′), 6.42 (s, 1H, H-4′), 6.96 (s, 1H, H-6′), 7.14 (m, 1H, H-2″), 6.98 (m, 1H, H-4″), 7.46 (m, 1H, H-5″), 7.18 (m, 1H, H-6″), 3.87 (s, 3H, 3″-OCH_3_). ^13^C NMR (100 MHz, Acetone-*d*_6_, ppm) δ 191.1 (C-1), 122.1 (C-2), 144.6 (C-3), 136.1 (C-1′), 107.0 (C-2′), 160.2 (C-3′), 107.2 (C-4′), 160.2 (C-5′), 107.0 (C-6′), 136.1 (C-1″), 116.2 (C-2″), 159.9 (C-3″), 113.7 (C-4″), 129.8 (C-5″), 121.2 (C-6″), 54.4 (3″-OCH_3_). HRMS (ESI, *m/z*) for C_16_H_14_O_4_ [M+H]^+^: calcd 270.2799, found 271.0976 (95).

#### 2.1.3. Synthesis of chalcone glycosides compounds (7a**–**c, 8a**–**c, 12a**–**c, 16a**–**c, and 17a**–**c)

To a solution of chalcones (1 mmol) (**6a–c, 11a–c,** and **15a–c**) in 10 mL anhydrous methanol, 5% KOH aqueous solution was stirred at 0–5 °C under nitrogen atmosphere, and then TAGBr (4.11 g, 1 mmol in 20 mL dry acetone) was added dropwise about 30 min. This reaction mixture was maintained continuously at 0–5 °C for 8 h. After 10 h at room temperature, the progress of the reaction was analyzed by TLC. The resultant solution was removed under reduced pressure. After evaporation, the brown syrupy was dissolved in AcOEt (20 mL), then neutralized with 5% HCl solution. The solution was extracted, dried over anhydrous Na_2_SO_4_, and concentrated in reduced pressure. The syrupy residue was purified using silica gel by column chromatography eluting with 20% MeOH in CHCl_3_ to yield acetylated chalcone glycosides (2,3,4,6-tetra-*O*-acetyl-*O*-β-glucopyranosyloxybenzlideneacetophenones).

Acetylated chalcone glycosides (0.1 g) were dissolved in 20 mL anhydrous methanol, and then stirred with freshly prepared sodium methoxide/methanol solution (5 mL, 0.05M) under nitrogen atmosphere. The reaction mixture was kept for 10 h at room temperature, then monitored the reaction progress using TLC. The mixture was then extracted with AcOEt (3 × 10 mL), dried over Na_2_SO_4_, concentrated in a rotary evaporator, and purified on silica gel using CHCl_3_-MeOH (10/- to 8/2, then 6/4, v/v) to afford the chalcone glycosides (**7a–c, 8a–c, 12a–c, 16a–c,** and **17a–c).** Compound **12a–c** were defined according to the previous studies [35–37].

##### 2.1.3.1. (2*E*)-1-(4-*O*-β-D-glucopyranosyl-3-methoxyphenyl)-3-(2-hydroxyphenyl)prop-2-en-1-one (7a)

Brown oil, yield: 78 mg (18%), IR *v_maks_*(cm^−1^): 3356 (-OH), 2949 (glucosidic C-H), 2839 (aliphatic C-H), 1647 (C=O), 1506, 1473 (C=C), 1016 (C-O), 651. ^1^H NMR (400 MHz, CD_3_OD, ppm) δ 7.80 (d, *J* = 15.7 Hz, 1H, H-2, A part of AB system), 8.18 (d, *J* = 15.7 Hz, 1H, H-3, B part of AB system), 7.45 (s, 1H, H-2′), 6.82 (d, *J* = 7.6 Hz, 1H, H-5′), 7.45 (d, *J* = 7.6 Hz, 1H, H-6′), 6.82 (m, 1H, H-3″), 7.45 (m, 1H, H-4″), 7.30 (m, 1H, H-5″), 7.80 (d, *J* = 7.8 Hz, 1H, H-6″), 3.85 (s, 3H, 3′-OCH_3_), 5.19 (d, *J* = 7.4 Hz, 1H), 3.0–3.8 (m, 4H), 3.90–4.14 (m, 2H). ^13^C NMR (100 MHz, CD_3_OD, ppm) δ 190.1 (C-1), 123.9 (C-2), 140.6 (C-3), 131.5 (C-1′), 111.4 (C-2′), 147.8 (C-3′), 152.2 (C-4′), 114.7 (C-5′), 120.8 (C-6′), 119.5 (C-1″), 157.5 (C-2″), 114.5 (C-3″), 132.6 (C-4″), 121.9 (C-5″), 129.1 (C-6″), 55.3 (3′-OCH_3_), 100.4 (β-anomeric carbon), 73.3 (C_2_-Glu), 76.4 (C_3_-Glu), 70.6 (C_4_-Glu), 76.7 (C_5_-Glu), 61.0 (C_6_-Glu). HRMS (ESI, *m/z*) for C_22_H_24_O_9_ [M+H]^+^: calcd 432.4205, found 433.1600 (20).

##### 2.1.3.2. (2*E*)-1-(4-*O*-β-D-glucopyranosyl-3-methoxyphenyl)-3-(3-hydroxyphenyl)prop-2-en-1-one (7b)

Brown oil, yield: 86 mg (20%), IR *v_maks_*(cm^−1^): 3358 (-OH), 2945 (glucosidic C-H), 2831 (aliphatic C-H), 1647 (C=O), 1456 (C=C), 1022 (C-O), 669. ^1^H NMR (400 MHz, Acetone-*d*_6_, ppm) δ 7.6 (d, *J* = 15.6 Hz, 1H, H-2, A part of AB system), 7.80 (d, *J* = 15.6 Hz, 1H, H-3, B part of AB system), 7.62 (s, 1H, H-2′), 7.0 (m, 1H, H-5′), 7.20 (m, 1H, H-6′), 6.78 (d, *J* = 2.8 Hz, 1H, H-2″), 7.10 (m, 1H, H-4″), 7.12 (m, 1H, H-5″), 7.10 (m, 1H, H-6″), 3.86 (s, 3H, 3′-OCH_3_), 5.14 (d, *J* = 7.6 Hz, 1H), 3.0–3.8(m, 4H), 3.90–4.10 (m, 2H). ^13^C-NMR (100 MHz, Acetone-*d*_6_, ppm) δ 187.5 (C-1), 123.5 (C-2), 143.6 (C-3), 132.3 (C-1′), 111.7 (C-2′), 149.5 (C-3′), 151.4 (C-4′), 115.9 (C-5′), 120.3 (C-6′), 136.9 (C-1″), 115.7 (C-2″), 157.7 (C-3″), 119.7 (C-4″), 121.7 (C-5″), 129.8 (C-6″), 55.6 (3′-OCH_3_), 100.4 (β-anomeric carbon), 73.6 (C_2_-Glu), 77.4 (C_3_-Glu), 70.1 (C_4_-Glu), 77.5 (C_5_-Glu), 61.4 (C_6_-Glu). HRMS (ESI, *m/z*) for C_22_H_24_O_9_ [M+H]^+^: calcd 432.4205, found 433.1589 (45).

##### 2.1.3.3. (2E)-1-(4-O-β-D-glucopyranosyl-3-methoxyphenyl)-3-(4-hydroxyphenyl)prop-2-en-1-one (7c)

Brown oil, yield: 108 mg (25%), IR *v_maks_*(cm^−1^): 3356 (-OH), 2949 (glucosidic C-H), 2839 (aliphatic C-H), 1635 (C=O), 1506, 1456 (C=C), 1014 (C-O), 669.^1^H NMR (400 MHz, Acetone-*d*_6_, ppm) δ 7.32 (d, *J* = 15.4 Hz, 1H, H-2, A part of AB system), 7.66 (d, *J* = 15.4 Hz, 1H, H-3, B part of AB system), 7.80 (s, 1H, H-2′), 7.40 (m, 1H, H-5′), 7.60 (m, 1H, H-6′), 7.70 (d, *J* = 7.8 Hz, 1H, H-2″), 6.86 (d, *J* = 7.8 Hz, 1H, H-3″), 6.86 (d, *J* = 7.8 Hz, 1H, H-5″), 7.70 (d, *J* = 7.8 Hz, 1H, H-6″), 3.87 (s, 3H, 3′-OCH_3_), 5.14 (d, *J* = 7.6 Hz, 1H), 3.0–4.0 (m, 6H). ^13^C NMR (100 MHz, Acetone-*d_6_*, ppm) δ 190.1 (C-1), 124.2 (C-2), 145.4 (C-3), 130.1 (C-1′), 113.6 (C-2′), 145.4 (C-3′), 151.8 (C-4′), 115.6 (C-5′), 123.8 (C-6′), 128.4 (C-1″), 132.4 (C-2″), 116.6 (C-3″), 161.7 (C-4″), 116.6 (C-5″), 132.4 (C-6″), 57.3 (3′-OCH_3_), 102.4 (β-anomeric carbon), 72.1 (C_2_-Glu), 78.9 (C_3_-Glu), 72.0 (C_4_-Glu), 75.5 (C_5_-Glu), 63.4 (C_6_-Glu). HRMS (ESI, *m/z*) for C_22_H_24_O_9_ [M+H]^+^: calcd 432.4205, found 433.1598 (15).

##### 2.1.3.4. (2*E*)-1-(4-O-β-D-glucopyranosyl-3-methoxyphenyl)-3-(2-*O*-β-D-glucopyranosyl phenyl)-prop-2-en-1-one (8a)

Orange oil, 59 mg (10%), IR *v_maks_*(cm^−1^): 3419 (-OH), 2924 (glucosidic C-H), 2856 (aliphatic C-H), 1734 (C=O), 1456 (C=C), 1234, 1033 (C-O), 759, 648. ^1^H NMR (400 MHz, CD_3_OD, ppm) δ 7.60 (d, *J* = 15.7 Hz, 1H, H-2, A part of AB system), 8.16 (d, *J* = 15.7 Hz, 1H, H-3, B part of AB system), 7.40 (s, 1H, H-2′), 6.90 (m, 1H, H-5′), 7.62 (m, 1H, H-6′), 6.98 (m, 1H, H-3″), 7.30 (m, 1H, H-4″), 7.20 (m, 1H, H-5″), 7.70 (m, 1H, H-6″), 3.75 (s, 3H, 3′-OCH_3_), 5.05 (d, *J* = 7.5 Hz, 1H), 5.08 (d, *J* = 7.7 Hz, 1H), 2.90–4.10 (m, 12H). ^13^C NMR (100 MHz, CD_3_OD, ppm) δ 190.1 (C-1), 123.9 (C-2), 140.6 (C-3), 131.6 (C-1′), 111.4 (C-2′), 150.8 (C-3′), 156.7 (C-4′), 115.6 (C-5′), 120.8 (C-6′), 124.6 (C-1″), 157.5 (C-2″), 114.5 (C-3″), 129.9 (C-4″), 121.8 (C-5″), 128.0 (C-6″), 55.2 (3′-OCH_3_), carbons of glucose-I and glucose-II (101.0, 100.4, 77.0, 76.9, 76.6, 76.4, 73.6, 73.5, 73.3, 69.9, 61.07, 61.06). HRMS (ESI, *m/z*) for C_28_H_34_O_14_ [M+2HOAc-OH-H]^+^: calcd 697.6577, found 697.3020 (80).

##### 2.1.3.5. (2*E*)-1-(4-*O*-β-D-glucopyranosyl-3-methoxyphenyl)-3-(3-*O*-β-D-glucopyranosyl phenyl)-prop-2-en-1-one (8b)

Yellow oil, 89 mg (15%), IR *v_maks_*(cm^−1^): 3374 (-OH), 2923 (glucosidic C-H), 1736 (C=O), 1582, 1419 (C=C), 1266, 1075 (C-O), 774, 674. ^1^H NMR (400 MHz, CD_3_OD, ppm) δ 7.70 (d, *J* = 15.4 Hz, 1H, H-2, A part of AB system), 7.86 (d, *J* = 15.4 Hz, 1H, H-3, B part of AB system), 7.72 (s, 1H, H-2′), 7.14 (s, 1H, H-5′), 7.30 (m, 1H, H-6′), 7.20 (m, 1H, H-2″), 6.90 (m, 1H, H-4″), 7.30 (m, 1H, H-5″), 7.18 (m, 1H, H-6″), 3.87 (s, 3H, 3′-OCH_3_), 5.09 (d, *J* = 7.4 Hz, 2H), 3.00–4.10(m, 12H). ^13^C-NMR (100 MHz, CD_3_OD, ppm) δ 189.3 (C-1), 123.1 (C-2), 144.6 (C-3), 136.2 (C-1′), 111.4 (C-2′), 149.4 (C-3′), 151.1 (C-4′), 114.7 (C-5′), 129.7 (C-6′), 132.3 (C-1″), 114.4 (C-2″), 157.9 (C-3″), 117.6 (C-4″), 132.3 (C-5″), 119.7 (C-6″), 55.9 (3′-OCH_3_), carbons of glucose-I and glucose-II (101.0, 100.4, 76.9, 76.6, 76.5, 73.6, 71.0, 70.2, 69.8, 61.3, 61.1). HRMS (ESI, *m/z*) for C_28_H_34_O_14_ [M+2HOAc-OH-H]^+^: calcd 697.6577, found 697.3038 (90).

##### 2.1.3.6. (2*E*)-1-(4-*O*-β-D-glucopyranosyl-3-methoxyphenyl)-3-(4-*O*-β-D-glucopyranosyl phenyl)-prop-2-en-1-one (8c)

Orange oil, 107 mg (18%), IR *v_maks_*(cm^−1^): 3376 (-OH), 2924 (glucosidic C-H), 2853 (aliphatic C-H), 1736 (C=O), 1583, 1456 (C=C), 1217, 1118 (C-O), 971, 771. ^1^H NMR (400 MHz, Acetone-*d*_6_, ppm) δ 7.58 (d, *J* = 15.8 Hz, 1H, H-2, A part of AB system), 7.66 (d, *J* = 15.8 Hz, 1H, H-3, B part of AB system), 7.58 (s, 1H, H-2′), 7.05 (s, 1H, H-5′), 7.64 (m, 1H, H-6′), 7.54 (m, 1H, H-2″), 7.12 (m, 1H, H-3″), 7.12 (m, 1H, H-5″), 7.54 (m, 1H, H-6″), 3.84 (s, 3H, 3′-OCH_3_), 4.98 (d, *J* = 7.4 Hz, 1H), 3.20–4.00 (m, 12H). ^13^C NMR (100 MHz, Acetone-*d*_6_, ppm) δ 189.2 (C-1), 123.9 (C-2), 144.5 (C-3), 136.2 (C-1′), 114.7 (C-2′), 149.4 (C-3′), 157.7 (C-4′), 117.4 (C-5′), 121.8 (C-6′), 132.3 (C-1″), 129.8 (C-2″), 114.7 (C-3″), 158.2 (C-4″), 114.7 (C-5″), 129.8 (C-6″), 55.2 (3′-OCH_3_), carbons of glucose-I and glucose-II (101.0, 100.4, 76.9, 76.4, 76.0, 73.6, 73.3, 70.0, 69.8, 69.7, 61.2, 61.0). HRMS (ESI, *m/z*) for C_28_H_34_O_14_ [M+2HOAc-OH-H]^+^: calcd 697.6577, found 697.3044 (35).

##### 2.1.3.7. (2*E*)-1-(3-*O*-β-D-glucopyranosyl-5-hyroxyphenyl)-3-(2-methoxyphenyl)-prop-2-en-1-one (16a)

Orange oil, 108 mg (25%), IR *v_maks_*(cm^−1^): 3377 (-OH), 2964 (glucosidic C-H), 2875, 2841 (aliphatic C-H), 1653 (C=O), 1587, 1456 (C=C), 1018 (C-O), 657. ^1^H NMR (400 MHz, Acetone-*d*_6_, ppm) δ 7.60 (d, *J* = 15.6 Hz, 1H, H-2, A part of AB system), 8.12 (d, *J* = 15.6 Hz, 1H, H-3, B part of AB system), 7.10 (s, 1H, H-2′), 6.80 (s, 1H, H-4′), 7.30 (s, 1H, H-6′), 6.80 (d, *J* = 7.6 Hz, 1H, H-3″), 7.00 (t, *J* = 7.4 Hz, 1H, H-4″), 7.40 (t, *J* = 7.4 Hz, 1H, H-5″), 7.92 (d, *J* = 7.6 Hz, 1H, H-6″), 3.86 (s, 3H, 2″-OCH_3_), 5.02 (d, *J* = 7.4 Hz, 1H), 3.0–4.0 (m, 6H). ^13^C NMR (100 MHz, Acetone-*d*_6_, ppm) δ 191.1 (C-1), 123.3 (C-2), 140.1 (C-3), 140.0 (C-1′), 107.8 (C-2′), 158.6 (C-3′), 111.1 (C-4′), 159.0 (C-5′), 109.1 (C-6′), 132.1 (C-1″), 158.9 (C-2″), 108.3 (C-3″), 128.6 (C-4″), 121.8 (C-5″), 120.6 (C-6″), 54.8 (2″-OCH_3_), 100.9 (β-anomeric carbon), 70.0 (C_2_-Glu), 76.5 (C_3_-Glu), 73.5 (C_4_-Glu), 76.8 (C_5_-Glu), 61.0 (C_6_-Glu). HRMS (ESI, *m/z*) for C_22_H_24_O_9_ [M+H]^+^: calcd 432.4206, found 433.1573 (35).

##### 2.1.3.8. (2*E*)-1-(3-*O*-β-D-glucopyranosyl-5-hyroxyphenyl)-3-(3-methoxyphenyl)-prop-2-en-1-one (16b)

Orange oil, 95 mg (22%), IR *v_maks_*(cm^−1^): 3356 (-OH), 2945 (glucosidic C-H), 2835 (aliphatic C-H), 1647 (C=O), 1541, 1471 (C=C), 1016 (C-O), 669. ^1^H NMR (400 MHz, Acetone-*d*_6_, ppm) δ 7.30 (d, *J* = 15.4 Hz, 1H, H-2, A part of AB system), 7.60 (d, *J* = 15.4 Hz, 1H, H-3, B part of AB system), 7.20 (s, 1H, H-2′), 6.85 (s, 1H, H-4′), 7.40 (s, 1H, H-6′), 7.30 (s, 1H, H-2″), 7.02 (d, *J* = 7.8 Hz, 1H, H-4″), 7.40 (m, 1H, H-5″), 7.15 (m, 1H, H-6″), 3.84 (s, 3H, 3″-OCH_3_), 5.04 (d, *J* = 7.4 Hz, 1H), 3.0–4.0(m, 6H). ^13^C NMR (100 MHz, Acetone-*d*_6_, ppm) δ 190.4 (C-1), 123.0 (C-2), 145.8 (C-3), 137.7 (C-1′), 106.6 (C-2′), 160.9 (C-3′), 108.8 (C-4′), 161.9 (C-5′), 109.8 (C-6′), 137.7 (C-1″), 116.2 (C-2″), 159.6 (C-3″), 114.6 (C-4″), 131.7 (C-5″), 123.6 (C-6″), 56.6 (3″-OCH_3_), 102.6 (β-anomeric carbon), 72.2 (C_2_-Glu), 78.6 (C_3_-Glu), 75.4 (C_4_-Glu), 78.7 (C_5_-Glu), 63.5 (C_6_-Glu). HRMS (ESI, *m/z*) for C_22_H_24_O_9_ [M+H]^+^: calcd 432.4206, found 433.1554 (90).

##### 2.1.3.9. (2*E*)-1-(3-*O*-β-D-glucopyranosyl-5-hydroxyphenyl)-3-(4-methoxyphenyl)-prop-2-en-1-one (16c)

Orange oil, 121 mg (28%), IR *v_maks_*(cm^−1^): 3377 (-OH), 2937 (glucosidic C-H), 2845 (aliphatic C-H), 1647 (C=O), 1568, 1510 (C=C), 1172, 1074, 1024 (C-O), 825, 790. ^1^H NMR (400 MHz, DMSO-*d*_6_, ppm) δ 7.60 (d, *J* = 15.4 Hz, 1H, H-2, A part of AB system), 7.62 (d, *J* = 15.4 Hz, 1H, H-3, B part of AB system), 7.20 (s, 1H, H-2′), 6.70 (s, 1H, H-4′), 7.30 (s, 1H, H-6′), 7.82 (d, *J* = 8.8 Hz, 1H, H-2″), 6.90 (d, *J* = 8.8 Hz, 1H, H-3″), 6.90 (d, *J* = 8.8 Hz, 1H, H-5″), 7.82 (d, *J* = 8.8 Hz, 1H, H-6″), 3.82 (s, 3H, 4″-OCH_3_), 5.08 (d, *J* = 7.5 Hz, 1H), 3.0–4.0 (m, 6H), 4.80–5.20 (m, 4OH). ^13^C NMR (100 MHz, DMSO-*d*_6_, ppm) δ 190.7 (C-1), 119.1 (C-2), 145.1 (C-3), 140.2 (C-1′), 107.7 (C-2′), 158.6 (C-3′), 108.3 (C-4′), 162.1 (C-5′), 109.0 (C-6′), 127.9 (C-1″), 130.3 (C-2″), 113.3 (C-3″), 159.0 (C-4″), 113.3 (C-5″), 136.3 (C-6″), 54.5 (4″-OCH_3_), 100.9 (β-anomeric carbon), 70.0 (C_2_-Glu), 76.5 (C_3_-Glu), 73.5 (C_4_-Glu), 76.9 (C_5_-Glu), 61.1 (C_6_-Glu). HRMS (ESI, *m/z*) for C_22_H_24_O_9_ [M+H]^+^: calcd 432.4206, found 433.1537 (75).

##### 2.1.3.10. (2*E*)-1-(3,5-di-*O*-β-D-glucopyranosylphenyl)-3-(2-methoxyphenyl)-prop-2-en-1-one (17a)

Brown oil, 148 mg (25%), IR *v_maks_* (cm^−1^): 3376 (-OH), 2980 (glucosidic C-H), 2864, 2825 (aliphatic C-H), 1734 (C=O), 1541, 1473 (C=C), 1238, 1031 (C-O), 669 (Aromatic stretching band). ^1^H NMR (400 MHz, CD_3_OD-*d*_4_, ppm) δ 7.70 (d, *J* = 15.7 Hz, 1H, H-2, A part of AB system), 8.20 (d, *J* = 15.7 Hz, 1H, H-3, B part of AB system), 7.20 (s, 1H, H-2′), 6.76 (s, 1H, H-4′), 7.30 (s, 1H, H-6′), 7.20 (m, 1H, H-3″), 7.12 (m, 1H, H-4″), 7.42 (t, *J* = 7.8 Hz, 1H, H-5″), 7.60 (d, *J* = 7.8 Hz, 1H, H-6″), 3.87 (s, 3H, 2″-OCH_3_), 5.0 (d, *J* = 7.4 Hz, 2H), 3.00–4.20 (m, 12H). ^13^C NMR (100 MHz, CD_3_OD-*d*_4_, ppm) δ 191.0 (C-1), 123.3 (C-2), 140.1 (C-3), 139.0 (C-1′), 111.2 (C-2′), 158.6 (C-3′), 109.0 (C-4′), 158.6 (C-5′), 111.2 (C-6′), 124.2 (C-1″), 158.9 (C-2″), 119.7 (C-3″), 128.7 (C-4″), 120.5 (C-5″), 132.0 (C-6″), 55.9 (2″- OCH_3_), carbons of glucose-I and glucose-II (103.9, 100.7, 76.4, 76.3, 74.6, 73.8, 73.5, 73.4, 70.3, 70.1, 63.4, 63.3). HRMS (ESI, *m/z*) for C_28_H_34_O_14_ [M-2MeOH+K+2H]^+^: calcd 571.5916, found 571.1341 (90).

##### 2.1.3.11. (2*E*)-1-(3,5-di-*O*-β-D-glucopyranosylphenyl)-3-(3-methoxyphenyl)-prop-2-en-1-one (17b)

Brown oil, 118 mg (20%), IR *v_maks_*(cm^−1^): 3372 (-OH), 2921 (glucosidic C-H), 2852 (aliphatic C-H), 2241, 1733 (C=O), 1591, 1451 (C=C), 1217, 1118 (C-O), 971. ^1^H NMR (400 MHz, CD_3_OD-*d*_4_, ppm) δ 7.60 (d, *J* = 15.7 Hz, 1H, H-2, A part of AB system), 7.76 (d, *J* = 15.7 Hz, 1H, H-3, B part of AB system), 7.20 (s, 1H, H-2′), 6.70 (s, 1H, H-4′), 7.30 (s, 1H, H-6′), 7.40 (s, 1H, H-2″), 7.02 (m, 1H, H-4″), 7.50 (m, 1H, H-5″), 7.36 (m, 1H, H-6″), 3.87 (s, 3H, 3″-OCH_3_), 4.98 (d, *J* = 7.4 Hz, 2H), 2.90–4.40 (m, 12H). ^13^C NMR (100 MHz, CD_3_OD-*d*_4_, ppm) δ 190.4 (C-1), 121.9 (C-2), 144.8 (C-3), 139.8 (C-1′), 112.9 (C-2′), 158.9 (C-3′), 109.2 (C-4′), 158.9 (C-5′), 112.9 (C-6′), 136.1 (C-1″), 116.4 (C-2″), 160.2 (C-3″), 116.4 (C-4″), 129.7 (C-5″), 121.9 (C-6″), 55.8 (3″- OCH_3_), carbons of glucose-I and glucose-II (103.9, 100.7, 76.4, 76.3, 74.0, 73.8, 73.6, 73.4, 70.3, 70.1, 63.4, 63.3). HRMS (ESI, *m/z*) for C_28_H_34_O_14_ [M-2MeOH+K+2H]^+^: calcd 571.5916, found 571.1358(40).

##### 2.1.3.12. (2*E*)-1-(3,5-di-*O*-β-D-glucopyranosylphenyl)-3-(4-methoxyphenyl)-prop-2-en-1-one (17c)

Brown oil, 160 mg (27%), IR *v_maks_*(cm^−1^): 3357 (-OH), 2973 (glucosidic C-H), 2927 (aliphatic C-H), 1738 (C=O), 1584, 1424 (C=C), 1172, 1044 (C-O), 879. ^1^H NMR (400 MHz, CD_3_OD-*d*_4_, ppm) δ 7.50 (d, *J* = 15.6 Hz, 1H, H-2, A part of AB system), 8.10 (d, *J* = 15.6 Hz, 1H, H-3, B part of AB system), 7.20 (s, 1H, H-2′), 6.75 (s, 1H, H-4′), 7.30 (s, 1H, H-6′), 7.90 (d, *J* = 7.8 Hz, 1H, H-2″), 6.90 (d, *J* = 7.8 Hz,1H, H-3″), 6.90 (d, *J* = 7.8 Hz,1H, H-5″), 7.90 (d, *J* = 7.8 Hz, 1H, H-6″), 3.86 (s, 3H, 4″-OCH_3_), 4.98 (d, *J* = 7.4 Hz, 2H), 2.90–4.30 (m, 12H). ^13^C NMR (100 MHz, CD_3_OD-*d*_4_, ppm) δ 189.1 (C-1), 121.8 (C-2), 143.5 (C-3), 138.6 (C-1′), 117.5 (C-2′), 157.4 (C-3′), 107.7 (C-4′), 157.4 (C-5′), 117.5 (C-6′), 125.8 (C-1″), 129.7 (C-2″), 111.6 (C-3″), 160.5 (C-4″), 11.67 (C-5″), 129.7 (C-6″), 54.3 (4″- OCH_3_), carbons of glucose-I and glucose-II (102.4, 99.4, 75.2, 75.0, 74.9, 72.0, 71.9, 68.6, 68.3, 68.2, 59.7, 59.5). HRMS (ESI, *m/z*) for C_28_H_34_O_14_ [M+2HOAc-OH]^+^: calcd 697.6577, found 697.3073 (50).

### 2.2. α-Glycosidase inhibitory effect

α-glycosidase inhibitory effect of the newly synthesized samples that were solvated in DMSO was performed with some minor modifications of the standard method [38]. The activity degree of the enzyme solution was adjusted as 2 U/mL in phosphate buffer (pH 6.8, 50 mM). For each test tube, 150 μL of the sample, 150 μL of the enzyme (2 U/mL), and 150 μL of buffer were pipetted. After the incubation for 15 min at 37 °C, 150 μL of *p*-nitrophenyl-α-D glucopyranoside (20 mM, Sigma-Aldrich) was added and then their absorbance was monitored for 20 min at 400 nm. Including the result of the positive control (Acarbose) known as the standard inhibitor, all results were given as IC_50_ value that means the concentration of the compound of giving 50% inhibition of maximal activity.

### 2.3. Tyrosinase from mushroom inhibitory effect

The tyrosinase inhibitory effects of the newly synthesized compounds were performed using microplate reader with minor modifications according to the standard method [39]. In this work, kojic acid was used as a standard compound and DMSO (%1) was used as the negative control. The activity of tyrosinase solution was adjusted as 250 U/mL in phosphate buffer (pH 6.8, 100 mM). For each wells, 100 μL buffer (pH 6.8, 100 mM), 20 μL of tyrosinase (0.2 U/mL), and 20 μL of compounds were added and incubated 20 min. After incubation, 20 μL of 3,4-dihydroxy-L-phenylalanine (3 mM) was pipetted into wells. Absorbance was measured at 475 nm. All results were given as IC_50_ value which means the concentration of the compound of giving 50% inhibition of maximal activity.

### 2.4. AChE from electric eel inhibitory effect

The AChE from electric eel inhibitory effects of the newly synthesized compounds were performed using microplate reader with minor modification according to the standard method [40]. In this work, galantamine was used as a standard compound and DMSO (%1) was used as negative control. The activity of AChE solution was adjusted as 0.2 U/mL in Tris-HCl buffer (pH 8.0, 50 mM). For each wells, 50 μL buffer (pH 8.0, 50 mM), 125 μL 5,5-dithio-bis(2-nitrobenzoic)acid (3 mM), 25 μL of AChE (0.2 U/mL) and 25 μL of compounds were added and incubated 20 min at room temperature. After incubation, 25 μL of acetylthiocholine iodide (15 mM) was pipetted into wells. Absorbance was measured at 412 nm. All results were given as IC_50_ value which means the concentration of the compound of giving 50% inhibition of maximal activity.

### 2.5. Molecular docking simulation

Molecular docking simulation was performed by AutoDock 4.2 [41] with the Lamanckian genetic algorithm and 100 run steps for each rigid target enzyme and a flexible compound. Since molecular docking is a structure-based drug design method, it requires a three-dimensional (3D) structural information of the target enzyme. Within this framework, the 3D crystal structures of target enzymes of α-glycosidase, tyrosinase, and AChE were accessed from the protein data bank website (http://www.rcsb.org/pdb) (PDB ID: 5NN4, 2Y9X, 4EY6 respectively). Water and ion molecules were removed from these 3D crystal structures, and appropriate hydrogen atoms were added under physiological pH conditions (pH = 7) using the APBS-PDB2PQR software [42]. The active site of target enzymes was determined by the AGFR1.2 [43] program according to the location of the binding site of the crystallized ligands. In accordance with this protocol, the binding free energy (ΔG) and inhibition constant (K_i_) values between the effector compounds that provide the most appropriate conformational fit to the 3D structure of the target enzymes were estimated.

## 3. Results and discussion

### 3.1. Chemistry

Scheme 2 displays the general synthetic route of the planned chalcone-*O*-glycosides (**7a–c, 8a–c, 12a–c, 16a–c,** and **17a–c**) in three steps. Firstly, a base-catalyzed Claisen-Schmidt reaction was applied between substituted acetophenones (**4**, **9**, and **13**) and substituted benzaldehydes (**5a–c**, **10a–c**, and **14a–c**), using 40% aq KOH in ethanol to yield the chalcone derivatives (**6a–c, 11a–c,** and **15a–c**) [12,44]. For *O*-glycosylation, TAGBr in dry acetone reacted with substituted chalcones (**6a–c, 11a–c,** and **15a–c**) using 5% aqueous KOH solution in methanol at 0–5 °C, to give acetyl chalcones [12,45]. Finally, the acetyl groups of compounds were carefully deprotected (Zemplen’s method) with methanolic NaOCH_3_ solution to yield the target chalcone-*O*-glycosides (**7a–c, 8a–c, 12a–c, 16a–c,** and **17a–c**) [45]. Compound **7c, 8c, 12a,** and **12c** was reported in earlier work [46,47]. The synthesis of the compounds **7a–b, 8a–b, 12b, 15a–b, 16a–c,** and **17a–c** are being declared for the first time. All synthesized compounds were well characterized by IR, NMR (^1^H and ^13^C), and mass spectrometry. In addition, all the obtained results were consistent with the expected structures for new chalcones **15a–b** and target chalcone-*O*-glycosides (**7a–c, 8a–c, 12a–c, 16a–c,** and **17a–c**) as displayed in Section 2.

The IR spectrum of **7a** possesses the characteristic band at 1647 cm^−1^, which indicates the –C=O group in the molecule. The strong absorption bands at *v*_maks_ 3356 and 1473 cm^−1^ indicated the existence of –OH and –C=C groups, respectively. Also, the IR spectra of compound **7a** showed a characteristic band assigning the glucosidic C-H at 2949 cm^−1^, which was confirmed by the presence of the *O*-β-D-glucosyl skeleton. The structures of synthesized compounds were further confirmed by their NMR (^1^H and ^13^C) spectrum, which revealed the signals of β or α glucosidic protons.

^1^H NMR spectrum of **7a** indicates two doublets at 7.80 ppm (*J* = 15.7 Hz, A moiety of AB system) and 8.18 ppm (*J*= 15.7 Hz, B moiety of AB system), indicating that the olefinic proton in the enone linkage is in the transconfiguration in the chalcone-*O*-glycoside. The transconfiguration structure (thermodynamically most stable) has been determined by this higher worth of coupling constant of olefinic protons [48,49]. As the proof of the chemical structure of chalcone-*O*-glycoside compounds (**7a–c, 8a–c, 12a–c, 16a–c,** and **17a–c**) showed a signal at δ 4.98–5.19 (d, approximately7.4 Hz) attributed to anomeric protons. That is also confirmed the β-orientation of the sugar unit. Moreover, β-glucopyranoside was approved to have a D-configuration from the positive valuation of the characteristic rotation after deacetylated compounds [50]. ^1^H NMR (400 MHz, DMSO-*d*_6_) spectrum of **16c** shows multiplet signals (5H) around δ 3.0–4.0 ppm assignable for the glucosidic proton of the chalcone-*O*-glycoside beside the signals corresponding to the glucosidic OH in the region δ 4.80–5.20 ppm (see Supplementary Information (SI)). Besides, the (**17a**) exhibited ^1^H NMR peaks at δ 5.00 (d, *J* = 7.4 Hz, 2H) and 3.00–4.20 (m, 10H, glucosidic proton) ppm indicating the linkage glucopyranosyl ring unit to the C-3″ and C-5″ position (see SI). As well, the ^1^H NMR spectra of compounds (**7a–c, 8a–c, 12a–c, 16a–c,** and **17a–c**) show a sharp singlet at δ 3.75–3.87 ppm due to OCH_3_ protons. The characteristic aromatic ring protons were located at the expected chemical shifts and integral values.

As well, we illustrate the behavior of ortho-OH substituted chalcones (**11a–c**) mono- and di-glycoside conjugates. At this juncture, we exploited chalcones (**11a–c**) as a starting materials unit to only mono-glucoside, which regarded chelate ring formation in the molecules. For example, the ^1^H NMR spectrum of **12c** shows one singlet characteristic peak around δ 12 ppm assignable for exchangeable OH proton of the chalcone-*O*-glycoside (see ESI).

^13^C NMR spectrum of **7a,** the characteristic signal for carbonyl carbon appeared at δ 190.1 (C-1). The same compound (**7a**) revealed signals assignable for olefinic carbons at 123.9 and 140.6 ppm. The ^13^C NMR spectrum of the chalcone-*O*-glycosides (**7a–c, 8a–c, 12a–c, 16a–c,** and **17a–c)** showed new signals assignable for anomeric carbons at around 100.0–103.9 ppm. The glycosidic bond of chalcone-*O*-glycoside was confirmed as *O*-glycosidic β-configuration [12,50–51]. Furthermore, ^13^C NMR spectrum of **17a** revealed signals of two *O*-glycoside groups at δ 100.7 and 103.9 ppm in addition to signals for di-glycoside moiety in their specific position (see ESI) [12,50,52]. The carbon signal of the methoxy groups was visible at δ approximately 55.0 ppm. ^13^C NMR spectra of chalcone-*O*-glycoside compounds show aromatic carbon peaks in the region δ 107.7–167.9 ppm.

Additionally, characteristic peaks were appeared in the mass spectra of chalcone-*O*-glycosides by the molecular ion peak at the *m*/*z* values, which confirmed their molecular mass. Compound **7c** as a brown oil, and its molecular formula founded as C_22_H_24_O_9_ from HRMS for the peak at *m*/*z* 433.1598 [M+H]^+^ (calc. for C_22_H_24_O_9_,432.4205). Compound **17a** as an brown oil, and its molecular formula established as C_28_H_34_O_14_ from HRMS for the peak at *m*/*z* 571.1341 [M-2MeOH+K+2H]^+^ (calc. for C_28_H_34_O_14_, 571.5916) (see ESI).

### 3.2. Evaluation of enzyme inhibitory effect

#### 3.2.1. α-Glycosidase inhibitory effect

α-glycosidase inhibition activity of some newly synthesized chalcone-*O*-glycoside derivatives. Except for **7a–c** and **8a–c** which were not any determined inhibition activity ranging of the studied concentrations, the compounds which were tagged with **12a–c, 16a–c,** and **17a–c** showed various degrees of α-glycosidase inhibition activity. Their IC_50_ values causing 50% inhibition of the enzyme ranged between 1.50 ± 0.02 and 23.60 ± 0.90 mM which **12a** was the best. Chalcones generally show pharmacological properties [53]. However, as far as we know, there are few studies based on α-glycosidase inhibition of chalcones and their derivatives in the literature. Fandaklı et al. [54] synthesized some hydroxy and methoxy substituted new chalcone oximes and evaluated them in terms of α-glycosidase inhibition activity. The study results revealed that many of the synthesis compounds exhibited the α-glycosidase inhibition activity by exhibiting various IC_50_ values in the range of 1.61–25.55 μM [54]. Moreover, Mukhtar and coworkers (2021) proved the α-glycosidase inhibition effect with chalcones derivatives especially substituted hydroxy and chloro one (IC_50_ = 1.19 ± 0.19 μM) [55]. For this study, although the synthesis compounds were different from previous literature sources and had low inhibition effects, it was important to find different inhibition degrees to show the correlation between biological activity and the importance of the synthesis.

#### 3.2.2. AChE inhibitory effect

The AChE inhibitory effects of the compounds were performed with minor modification according to the standard method. The IC_50_ values of the compounds were given in Table 1. As shown in Table 1, the compounds had low inhibitory effects when compared to galantamine (IC_50_ = 20.10 ± 0.25 μM). The IC_50_ value of **8b** was determined as 81.79 ± 3.40 μM and it had the highest inhibitory effect among the tested compounds. The IC_50_ values of other compounds have higher than 100 μM (Table 1).

#### 3.2.3. Tyrosinase inhibitory effect

The tyrosinase inhibitory effects of the compounds were performed with minor modification according to the standard method. The IC_50_ values of the compounds were given in Table 1. **7c** had the highest inhibitory effects on tyrosinase with IC_50_ value of 11.07 ± 0.55 μM. **7c** showed approximately 2.76-fold more effective inhibitory properties than kojic acid against tyrosinase. The IC_50_ values of **17c**, **8c** and **7b** were found to be 34.42 ± 1.74, 53.55 ± 2.31, and 82.47 ± 4.50, respectively.

### 3.3. Molecular docking

**Figure 1:**
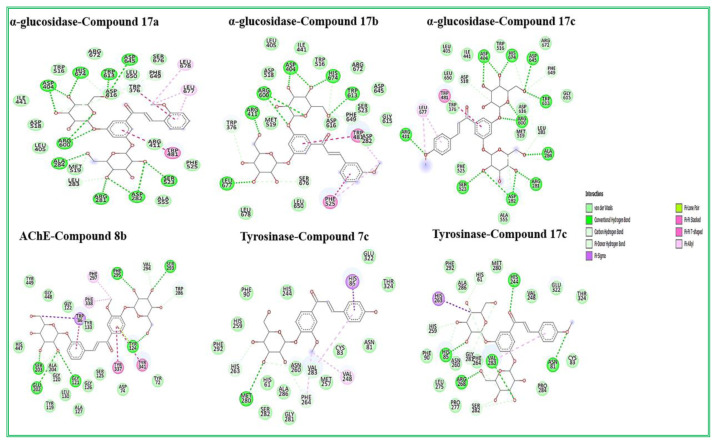
2D analysis of the lowest energy binding conformations of α-glycosidase, AChE and tyrosinase with the most effective compounds.

**Figure 2:**
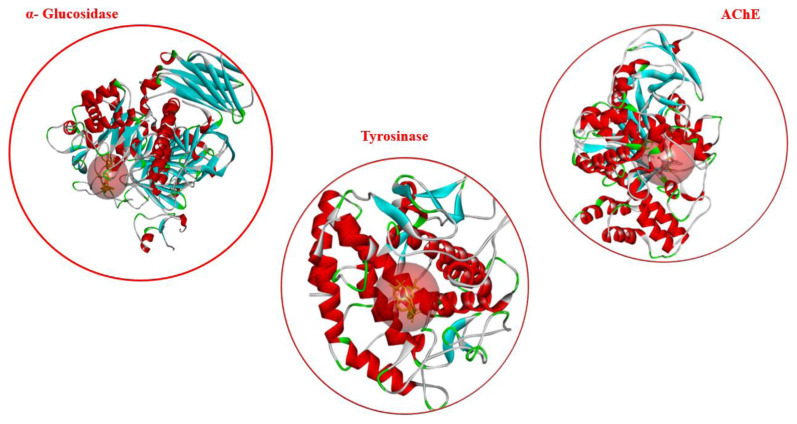
3D structures of α-glycosidase, AChE and tyrosinase and its binding site (red sphere).

In this study, molecular docking analysis was applied to elucidate the binding mechanism of α-glycosidase, tyrosinase and AChE and new chalcone-*O*-glycosides compounds. According to the molecular docking analysis for the AChE enzyme, all compounds exhibited better binding affinity than the reference compound galantamine (see in Table 2). Among these compounds, **17a**, **17b**, **17c,** and **8b** are the most effective compounds with the best lowest binding energy values (−13.73, −14.06, −13.37, −13.24 kcal/mol, respectively), also compound **8b** was found to be effective against AChE in experimental studies. The active site of AChE has the catalytic triad consisting of Ser203, His447, Glu334, and the anionic subsite consisting of Trp86, Glu202 and Tyr337 [56–57]. According to the docking pose of compound **8b**, which shows the highest inhibitory effect against AChE both experimental and docking studies, it was observed that bound with Gly121, Tyr124, Ser203, Phe295, Ser293, Glu202, Gly120, Val294, Trp286 in tyrosinase with hydrogen bonds (Figures 1 and 2). Besides, this compound formed π-sigma with Trp86, π-lone pair with Tyr124, π T-shaped with Tyr337 and Tyr341, π-alkyl with Phe297 and Phe338 in the AChE active site.

Furthermore, compounds **17a**, **17b**, and **17c** showed the best binding affinity against tyrosinase enzyme, compound **17c** also showed good inhibitory effects in experimental studies. The tyrosinase enzyme contains conserved histidine residues (His61, His85, His94, His259, His263, His269) in the active site. These residues are necessary for the tyrosinase enzyme catalytic activities [58]. According to the docking study, compound **17c**, which shows the effective compound against tyrosinase with experimental and docking studies, it formed hydrogen bond interactions with His85, His61 and His259 conserved histidine residues of tyrosinase enzyme active site. This compound also formed hydrogen bond interactions with Val283, Arg268, Asn81, Gly281, Ser282 located in the active site of tyrosinase (Figure 1). Similarly, compounds **17a**, **17b**, and **17c** also exhibited very strong binding affinity against the α-glycosidase enzyme with the best lowest binding energy values (−10.68, −10.30, −10.58 kcal/mol, respectively) (Table 2). These active compounds commonly formed hydrogen bonds with Arg600, Asp404, His674, Asp645, Ser523, and Asp282 residues in the α-glycosidase active site (Figure 1).

## 4. Conclusion

To sum up, mono- and di-glycoside conjugates of some novel chalcone-*O*-glycoside derivatives (**7a–c, 8a–c, 12a–c, 16a–c,** and **17a–c**) have been designed and synthesized. The chemical structures of all synthesized compounds were characterized by different spectroscopic methods. Among the entire nine chalcone compounds (**6a–c, 11a–c,** and **15a–c**), owing to the presence of chelate ring formation of ortho- position some chalcones (**11a–c**) are difficult to di-glycosylation by chemical methods. Chalcones and their derivatives have been a popular investigation area because of having potential enzyme inhibition activity in the literature. However, there are also deficiencies in terms of combinatorial studies. For this reason, this in situ study was designed and some *in vitro* enzyme (α-glycosidase, acetylcholinesterase, and tyrosinase) inhibitory activities of chalcone-*O*-glycoside derivatives were evaluated with molecular docking analysis for the first time. In particular, compound **7c** exerts the best potency (IC_50_ = 11.07 ± 0.55 μM) of tyrosinase inhibition, which is 2.76-fold lower than reference drug kojic acid (11.07 ± 0.55 μM). In addition, compound **17c** revealed good inhibition (IC_50_ = 34.42 ± 1.74 μM) for tyrosinase. Furthermore, for acetylcholine esterase inhibition, compound **8b** had a prominent value of 81.79 ± 3.40 μM among the others. According to the obtained data, it was seen that there was a low activity degree for α-glycosidase enzyme inhibition even though there was a nearly wide range. Here, **12a** is the most effective (IC_50_= 1.50 ± 0.02 mM), followed by **12b** (IC_50_ = 5.10 ± 0.06 mM), **16b** (IC_50_ = 6.60 ± 0.05 mM), and **16c** (IC_50_ = 6.80 ± 0.06 mM).

As it is known, molecular docking studies are a simulation of in vivo interactions and are evaluated under the categories of in vitro analyses. The data of them are theoretical and provide information about the binding activities and energies of the current enzyme and its substrate as ideal. According to molecular docking analysis, compounds **17a, 17b,** and **17c** showed very good binding affinities against all targeted enzymes. These results suggest that chalcone-*O*-glycoside derivatives could be synthesized for future studies that have different functional groups in each aromatic ring, thus possibly becoming a potential tyrosinase inhibition activity. In vitro enzyme inhibition findings could be anticipated to be consistent with probable molecular docking results; however, the results were not at the same level in repeated analyses under in vitro application analyses in the current study. In any study, if a significant correlation between in vitro application assays and molecular docking is observed, extremely easy and impressive evaluations can be made about synthesis compounds and their inhibition activities. However, if there is no correlation, compound evaluations related to the enzyme inhibition should be performed under different conditions. Because the enzymes and enzymatic studies are directly affected by many factors, such as heat, light, UV, incubation, the concentration of substrate, other chemical reagents, and the analyzer effect. This might be the main source of the inconsistency in the current results.

Figure 1 ^1^H data for compound **12c.**

Figure 2 ^1^H data for compound **16c.**

Figure 3 ^1^H, ^13^C and HRMS data for compound **17a.**

Figure 4 HRMS data for compound **7c.**

## Figures and Tables

**Table 1:** IC_50_ values of the compounds on *α*-glycosidase, tyrosinase, and AChE.

No	α-glycosidase (mM)	Tyr (μM)	AChE (μM)
**7a**	nd	>100	>100
**7b**	nd	82.47 ± 4.50	>100
**7c**	nd	11.07 ± 0.55	>100
**8a**	nd	>100	>100
**8b**	nd	>100	81.79 ± 3.40
**8c**	nd	53.55 ± 2.31	>100
**12a**	1.50 ± 0.02	>100	>100
**12b**	5.10 ± 0.06	>100	>100
**12c**	12.38 ± 0.10	>100	>100
**16a**	12.87 ± 0.09	>100	>100
**16b**	6.60 ± 0.05	>100	>100
**16c**	6.80 ± 0.06	>100	>100
**17a**	23.60 ± 0.90	>100	>100
**17b**	16.62 ± 0.62	>100	>100
**17c**	16.30 ± 0.63	34.42 ± 1.74	>100
Galantamine	-	-	20.10 ± 0.25
Kojic acid	-	30.59 ± 0.30	-
Acarbose (μM)	0.03 ± 0.00	-	-

nd: not detected.

**Table 2:** The lowest binding energy values of the chalcone-*O*-glycosides compounds and reference compounds from each docking analysis in the active site of α-glycosidase, tyrosinase, and AChE.

No	α-glycosidase binding energy (kcal/mol)	Tyrosinase binding energy (kcal/mol)	AChE binding energy (kcal/mol)
**7a**	−8.48	−7.97	−11.22
**7b**	−8.22	−8.18	−11.15
**7c**	−8.51	−8.01	−10.99
**8a**	−9.41	−7.41	−12.70
**8b**	−9.55	−6.91	−13.24
**8c**	−8.38	−7.08	−10.83
**12a**	−8.12	−7.92	−11.14
**12b**	−8.72	−8.22	−10.98
**12c**	−8.60	−8.10	−10.91
**16a**	−8.68	−8.13	−10.91
**16b**	−8.40	−8.60	−11.41
**16c**	−8.46	−8.66	−11.21
**17a**	−10.68	−9.47	−13.73
**17b**	−10.30	−10.20	−14.06
**17c**	−10.58	−10.49	−13.37
Galantamine	-	-	−9.13
Kojic acid	-	−3.96	-
Acarbose	−4.66	-	-
