# Supplementary figures and images for: Design, synthesis, and enzyme inhibition evaluation of some novel Mono- and Di-O-β-D-Glycopyranosyl Chalcone analogues with molecular docking studies
Source: Turk J Chem. 2022 Nov 23;47(1):171–84. doi: 10.55730/1300-0527.3527 (PMC10504015; doi:10.55730/1300-0527.3527)

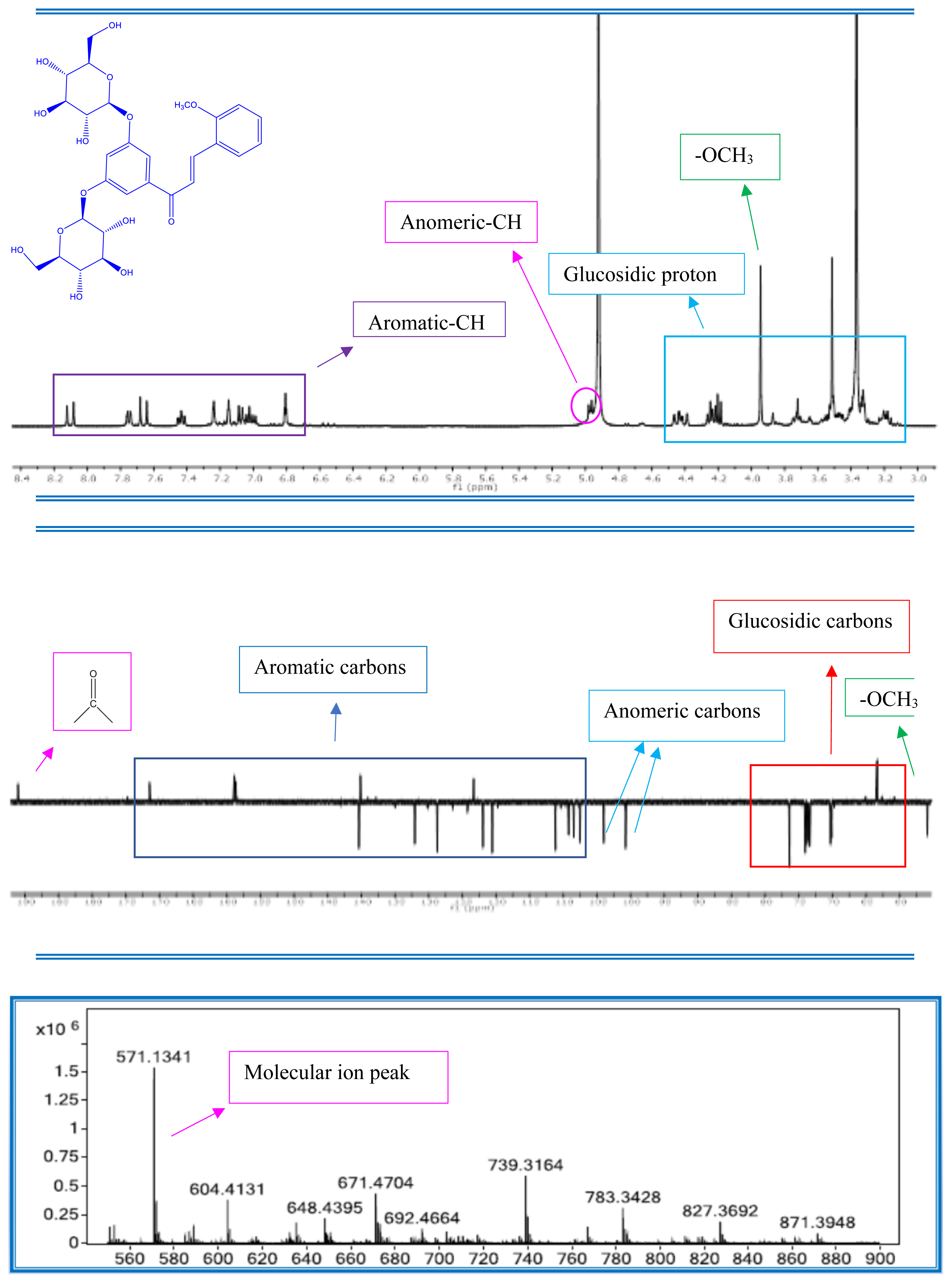

Supplement: Figure 1 — 1H data for compound 12c. [file turkjchem-47-1-171s1.tif]

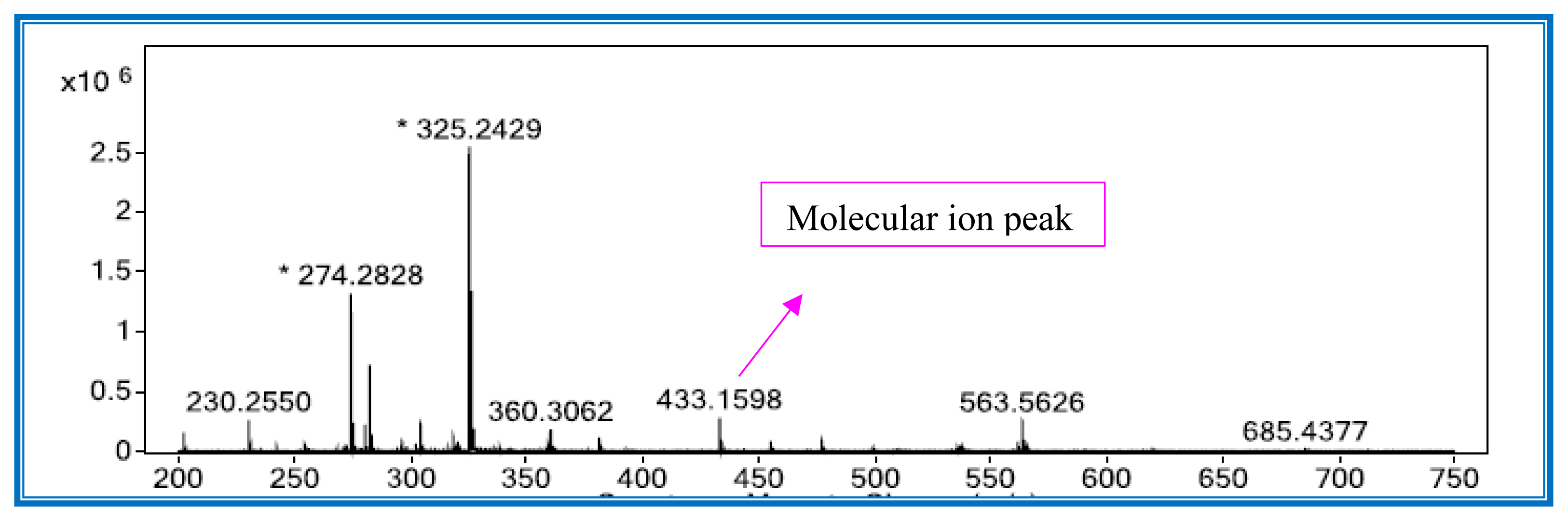

Supplement: Figure 2 — 1H data for compound 16c. [file turkjchem-47-1-171s2.tif]

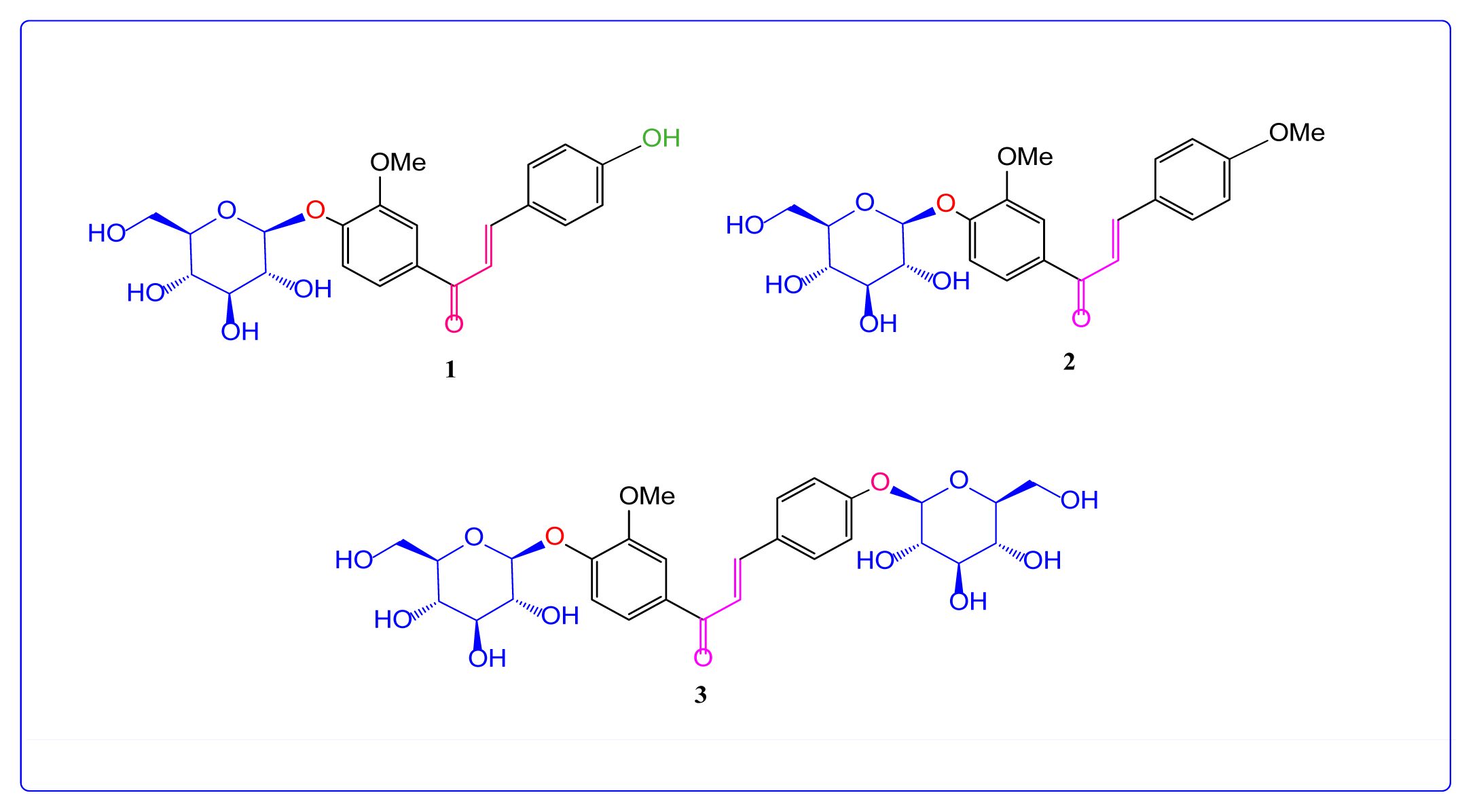

Supplement: Figure 3 — 1H, 13C and HRMS data for compound 17a. [file turkjchem-47-1-171s3.tif]

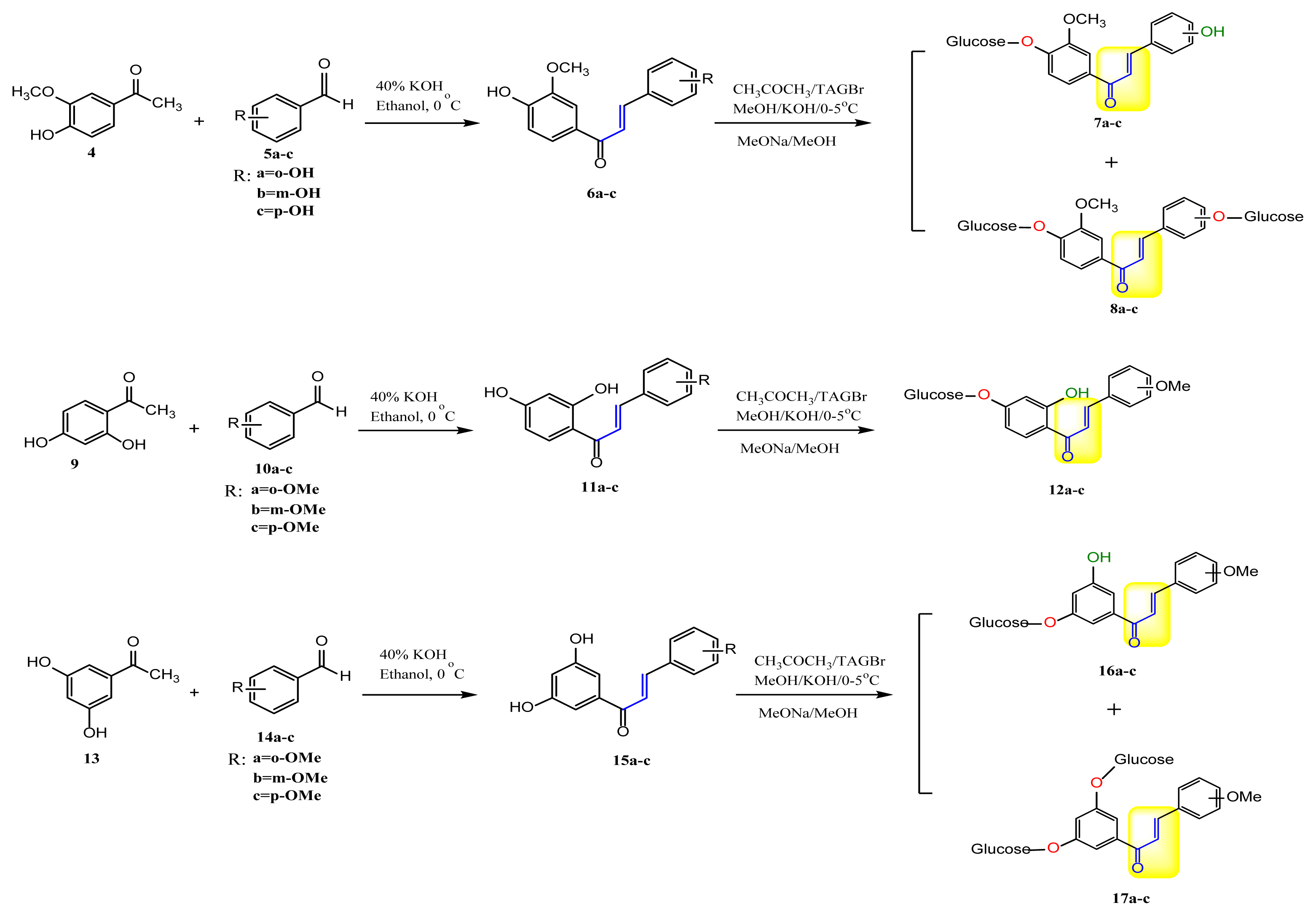

Supplement: Figure 4 — HRMS data for compound 7c. [file turkjchem-47-1-171s4.tif]
